# Retraction: Inhibition of the Receptor Tyrosine Kinase ROR1 by Anti-ROR1 Monoclonal Antibodies and siRNA Induced Apoptosis of Melanoma Cells

**DOI:** 10.1371/journal.pone.0268357

**Published:** 2022-05-05

**Authors:** The PLOS ONE Editors

Following the publication of this article [1], concerns were raised regarding results presented in Figures 5 and 6. Specifically,

- The Figure 5A ESTDAB049 and ESTDAB075 Actin panels appear similar.
- The following FACS panels in Figure 6 appear more similar than would be expected from independent samples:
  - The top and bottom left quadrants of the Untransfected ESTDAB049, Untransfected ESTDAB075, and Untransfected ESTDAB081 panels.
  - The top and bottom left quadrants of the Control siRNA ESTDAB049, Control siRNA ESTDAB075, and Control siRNA ESTDAB081 panels.
  - The bottom right quadrants of the Control siRNA ESTDAB075 and Control siRNA ESTDAB081 panels.
  - The Untransfected A375, Control siRNA A375, and siRNA treated A375 panels.
  - The top left quadrants of the Untransfected ESTDAB112 and Control siRNA ESTDAB112 panels.
  - The Control siRNA T47D and siRNA treated T47D panels.

Regarding Figure 5A, the corresponding author confirmed that the ESTDAB075 Actin panel was inadvertently duplicated and offered a replacement figure to correct the ESTDAB049 Actin panel.

Regarding Figure 6, the corresponding author stated that the ESTDAB cell lines are very similar to one another in size and granularity. Furthermore, the corresponding author stated that although parts of the panels appear similar, clear differences in apoptosis and necrosis can be seen between the untransfected, control siRNA, and siRNA transfected samples. The explanation provided does not clarify the concerns pertaining to the high level of similarity observed between multiple panels presented in this figure.

The corresponding author stated that the raw data underlying the results presented in Figures 5 and 6 are no longer available.

In light of the unresolved concerns with Figure 6 that question the validity and reliability of the reported results, the *PLOS ONE* Editors retract this article.

All authors disagree with the retraction decision.
